# Paradigm shift in macrophage polarization in osteoarthritis: from M1/M2 imbalance to macrophage state reprogramming in the ageing immune microenvironment

**DOI:** 10.3389/fimmu.2026.1942715

**Published:** 2026-09-04

**Authors:** Bandianzhuoma, Shibin Yang, Jianjiao Mou, Lianlin Zeng

**Affiliations:** 1 The University of Queensland, Brisbane, QLD, Australia; 2 Department of Rehabilitation Medicine, The Affiliated Hospital of Southwest Medical University, Luzhou, China; 3 Department of Rehabilitation Medicine, Suining Central Hospital, Suining, China

**Keywords:** ageing immune microenvironment, M1/M2 imbalance, macrophage polarization, macrophage reprogramming, osteoarthritis

## Abstract

Osteoarthritis (OA) is one of the most common degenerative joint diseases, and its initiation and progression are closely associated with the inflammatory microenvironment within the joint. As key effector cells of innate immunity in the joint, macrophages contribute to synovial inflammation, cartilage matrix degradation, and impaired tissue repair. Conventional studies have largely interpreted the immunopathology of OA through a binary model of macrophage polarization, in which pro-inflammatory M1 macrophages and anti-inflammatory M2 macrophages are considered opposing functional states. Within this framework, M1/M2 imbalance is viewed as a major mechanism driving persistent inflammation and cartilage destruction in OA. However, increasing evidence from studies of the ageing-associated immune microenvironment indicates that macrophage states in OA extend well beyond the classical M1/M2 classification. These states involve the senescence-associated secretory phenotype, functional remodelling of tissue-resident macrophages, metabolism-driven phenotypic transitions, and dynamic regulation through intercellular communication networks. This review summarizes the evidence supporting a conceptual transition from M1/M2 polarization imbalance to ageing immune microenvironment-driven macrophage state reprogramming in OA. We focus on the underlying molecular mechanisms, cellular interactions, and pathological implications, and further evaluate potential therapeutic strategies based on macrophage functional reprogramming, with the aim of providing a mechanistic and translational basis for precision interventions in OA.

## 1 Introduction

Osteoarthritis (OA) is a chronic degenerative disease that affects the entire joint organ, with pathological changes including cartilage degeneration, synovial inflammation, subchondral bone remodelling, and osteophyte formation. With population ageing and the increasing prevalence of obesity, OA has become a major contributor to pain, functional impairment, and reduced quality of life in middle-aged and older adults (1, 2). OA was traditionally regarded primarily as cartilage degeneration caused by mechanical wear. However, substantial evidence now indicates that low-grade chronic inflammation and immune dysregulation are involved in the onset and progression of OA (3, 4). Within the joint immune microenvironment, macrophages are key innate immune cells that maintain tissue homeostasis and mediate inflammatory responses, and their functional states have a substantial influence on OA pathogenesis (5, 6).

The M1/M2 framework remains a useful experimental shorthand for macrophage responses to defined stimuli. Interferon-γ, microbial products, and pro-inflammatory cytokines induce M1-like programmes marked by IL-1β, TNF-α, inducible nitric oxide synthase, and matrix metalloproteinases (MMPs), whereas IL-4/IL-13-associated M2-like programmes are linked to IL-10, transforming growth factor-β, arginase-1, and tissue repair (2, 6). This framework has guided therapies designed to suppress M1-like activity or enhance M2-like responses, including natural compounds, glucocorticoids, extracellular vesicles (EVs), and reactive oxygen species (ROS)-responsive nanomedicines (7–11). However, these programmes were largely defined under controlled *in vitro* conditions and should be treated as stimulus-dependent states rather than fixed lineages or mutually exclusive identities in diseased human joints.

Ageing makes this limitation clinically important. Within an aged joint, chronic SASP exposure, mitochondrial ROS and mitochondrial DNA release, altered glucose and lipid metabolism, extracellular matrix (ECM)-derived damage-associated molecular patterns, defective efferocytosis, and recurrent inflammatory stimulation may converge in the same macrophage. The resulting cells can co-express M1- and M2-associated markers, retain inflammatory or metabolic memory after the initiating signal has waned, or lose phagocytic and reparative competence without strongly expressing either marker set. Ontogeny adds another layer: long-lived tissue-resident macrophages and recruited monocyte-derived macrophages may respond differently to identical local cues. Marker-based M1/M2 assignment therefore cannot resolve state history, reversibility, metabolic fitness, efferocytic capacity, or anatomical compartment (12).

**Figure 1:**
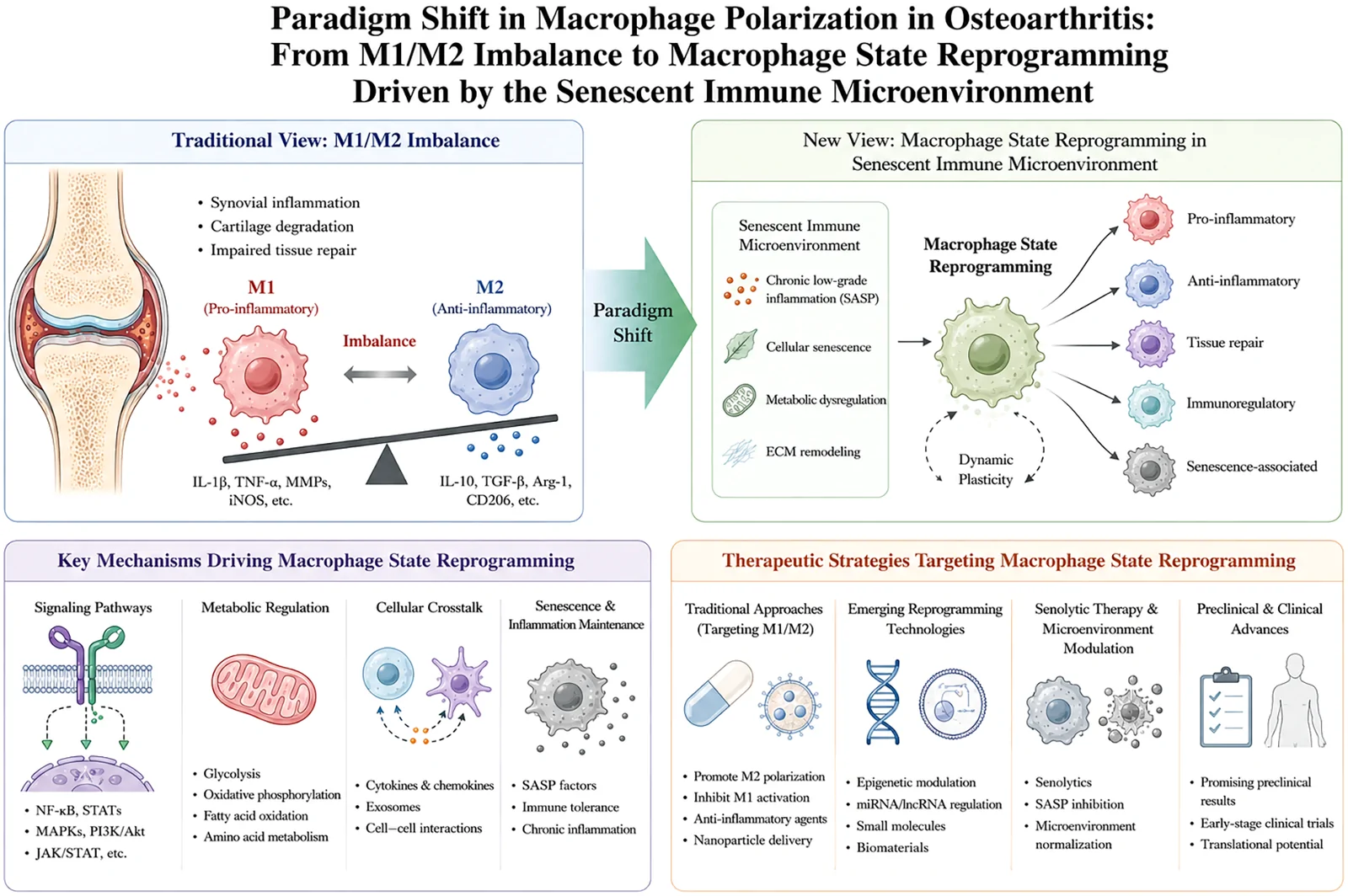
Conceptual shift from M1/M2 imbalance to macrophage state reprogramming in the ageing OA immune microenvironment. This schematic diagram was drawn by the author using BioRender (https://app.biorender.com) and polished by artificial intelligence.

Accordingly, the review first defines the experimental utility and ageing-specific limitations of classical polarization, then examines how senescence and immunosenescence alter macrophage states. We focused on emergent mechanisms that can stabilise or transmit state changes, including SASP-driven enhancer remodelling, mitochondrial dysfunction and trained innate memory, intercellular metabolic coupling, and macromolecular condensates. The final section critically evaluates whether therapies should repolarise macrophages directly, remove upstream senescent-cell signals, restore resolution functions, or combine these approaches. Recent research has therefore shifted towards mechanistic analyses of macrophage state reprogramming in the ageing immune microenvironment (**Figure 1**). This review discusses this paradigm shift in macrophage polarization in OA, with particular emphasis on the molecular mechanisms and therapeutic implications.

## 2 Macrophage polarization and M1/M2 imbalance in OA

### 2.1 Classical definition and functional differences of macrophage polarization

**Figure 2:**
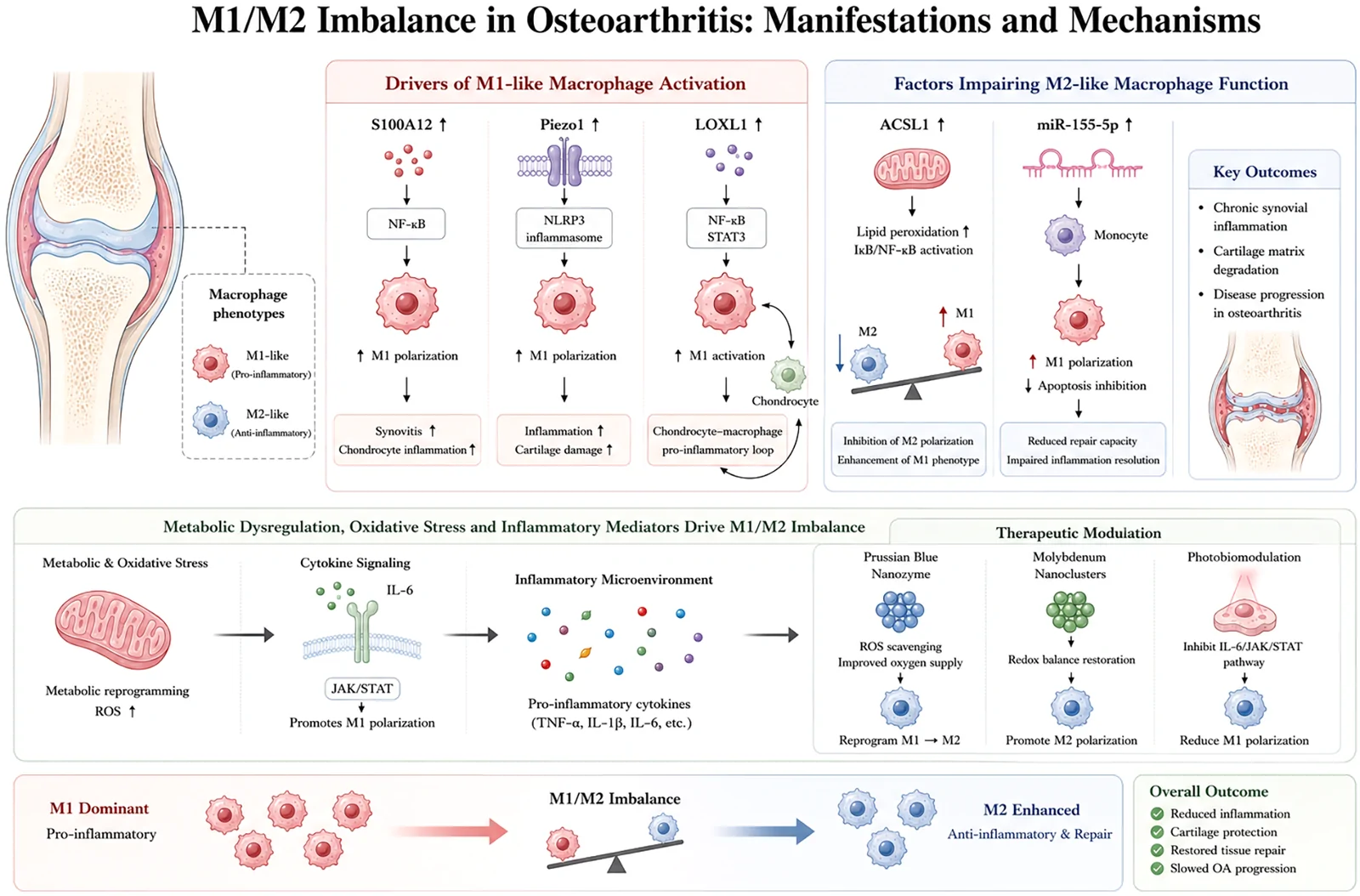
Manifestations and mechanisms of M1/M2 imbalance in OA, including inflammatory activation, impaired repair-associated function, metabolic dysregulation, and therapeutic modulation. This schematic diagram was drawn by the author using BioRender (https://app.biorender.com) and polished by artificial intelligence.

Macrophage polarization describes the capacity of macrophages to adopt stimulus- and context-dependent functional programmes. In OA, dysregulated activation of these programmes can amplify synovial inflammation, matrix degradation, and tissue damage (**Figure 2**) (6, 13, 14). The M1/M2 model provides a useful entry point for this biology, but it does not encompass the full range of macrophage states observed in diseased joints. Recent reviews similarly identify macrophage polarization as a therapeutic target while emphasising the need to interpret M1/M2 phenotypes within the wider OA microenvironment (15).

M1-like programmes are induced by pro-inflammatory stimuli through pathways that include NF-κB, MAPK, and JAK/STAT. They are commonly associated with CD86, inducible nitric oxide synthase, TNF-α, IL-1β, IL-12, and IL-18 (16–19). These mediators stimulate chondrocytes and synovial fibroblasts to increase MMPs and aggrecanases, thereby accelerating ECM degradation (18, 19). M1-like macrophage activity can also reinforce synovitis, osteoclastogenesis, and osteophyte formation (2). In particular, macrophage-derived IL-18 has been linked to chondrocyte senescence and cartilage injury (18, 19). These features describe a pro-inflammatory programme rather than a stable lineage.

M2-like programmes are associated with inflammation resolution and tissue repair. They commonly include CD206 and arginase-1 expression together with IL-10 and TGF-β production (19). In ageing-associated OA, several studies report an increase in M1-like macrophage features and a relative reduction in M2-like features across bone and cartilage compartments (20, 21). This shift can propagate through macrophage–fibroblast and macrophage–chondrocyte crosstalk, creating a self-reinforcing inflammatory circuit (22). Accordingly, interventions that favour repair-associated macrophage functions remain attractive (23, 24), but therapeutic success should be judged by durable resolution, efferocytosis, and structural protection rather than by surface markers alone.

### 2.2 Manifestations and mechanisms of M1/M2 imbalance in OA pathology

Clinical samples and experimental OA models commonly show enrichment of M1-like inflammatory macrophages, often in association with disease severity. S100A12 in OA synovial fluid can activate NF-κB and promote M1-like polarization, thereby aggravating synovitis and chondrocyte inflammation (25). In synovial macrophages, upregulated Piezo1 can activate the NLRP3 inflammasome and reinforce inflammatory polarization (26). Chondrocyte-derived lysyl oxidase-like 1 provides a complementary mechanism by activating macrophage NF-κB and STAT3 signalling, creating a bidirectional pro-inflammatory feedback loop (27). Thereby, these observations place macrophage activation within a multicellular network rather than an isolated polarization event.

Loss of repair and resolution functions is equally important. Long-chain acyl-CoA synthetase 1 is upregulated in OA synovial macrophages and shifts lipid metabolism towards peroxidative stress, suppressing M2-like polarization while enhancing IκB/NF-κB-dependent inflammatory features (28). OA synovial fluid can also drive M1-like polarization of peripheral-blood monocyte-derived macrophages through miR-155-5p and inhibit macrophage apoptosis (29). Thus, impaired M2-like function is not simply the reciprocal of M1 activation; it may independently compromise efferocytosis, matrix repair, and the termination of inflammation.

Redox imbalance and metabolic dysfunction are major determinants of macrophage state. Ultrasmall Prussian blue nanozymes scavenge ROS and improve oxygen availability, shifting macrophage readouts towards an M2-like profile and attenuating experimental OA (30). Molybdenum nanoclusters similarly restore redox balance and promote repair-associated polarization (31). These studies are consistent with a role for energy metabolism in macrophage activation, while photobiomodulation can reduce M1-like polarization by inhibiting IL-6/JAK/STAT signalling (28, 32). Nevertheless, changes in marker expression should not automatically be equated with durable state conversion; persistence after treatment withdrawal and restoration of macrophage function remain important endpoints.

### 2.3 Limitations of the classical M1/M2 polarization model

The M1/M2 model provides concise vocabulary but compresses the multi-dimensional state space into binary oppositions. Macrophage activation is continuous and includes intermediate or mixed-type processes, while the joints of osteoarthritis (OA) are simultaneously exposed to mechanical stress, matrix debris, cytokines, metabolites, and senescence-related signals (15, 33). Consistent with this complexity, CD163-positive OA synovial macrophages may simultaneously express CD80, CD206 and IL-10, and single-cell RNA sequencing also identified subpopulations enriched in phagocytic processes, growth factors and inflammatory programs, which do not fully fall into the M1 or M2 categories (12, 34). Therefore, the M1/M2 axis is best used as a descriptive dimension rather than a complete classification system.

Macrophage analysis in OA should therefore integrate cell origin, anatomical compartment, functional competence, and temporal trajectory. Single-cell transcriptomics can resolve previously unrecognised subsets and molecular programmes, and integration with bulk RNA sequencing can help relate these subsets to tissue-level disease signatures (34–36). Multiparameter flow cytometry adds protein-level phenotyping and functional validation (35, 36). When combined with lineage tracing, spatial methods, metabolic assays, and efferocytosis measurements, these approaches may support therapies directed at defined macrophage states rather than broad M1/M2 labels (37, 38).

### 2.4 Relevant signalling pathways and regulatory factors

Several recurrent pathways provide a baseline for interpreting macrophage responses in OA. NF-κB integrates inflammatory cytokines, matrix-derived danger signals, and mechanical stress, whereas PI3K-AKT, AMPK, Nrf2, and YAP-dependent pathways link survival, redox balance, metabolism, and tissue repair (39–41). STAT1- and STAT6-associated programmes bias inflammatory and type 2 responses, respectively, and experimental modulation of JAK/STAT signalling can alter macrophage polarization in OA (16, 17). Specific examples include inhibition of the GSK3β/NF-κB/CCL20 axis by betulinic acid, modulation of PI3K/AKT/NF-κB signalling by mechanical loading (42), and PI3K-AKT-dependent repair responses induced by human umbilical cord mesenchymal stromal-cell EVs (10, 43). These pathways overlap extensively and should not be assigned exclusively to M1 or M2 states.

MicroRNAs and EV cargo further tune this signalling network. miR-9-5p targets SIRT1 and alters NF-κB/AMPK activity (44); miR-155-5p regulates suppressor of cytokine signalling 1 and caspase-3 (29); hsa_circ_0005567 acts through the miR-492/SOCS2 axis (45); and EV-associated miR-122-5p influences PI3K-AKT signalling (10). Rather than recataloguing these canonical pathways, Section 3 focuses on mechanisms that could integrate or stabilise macrophage state over time: SASP-driven enhancer remodelling, mitochondrial dysfunction and trained innate memory, intercellular metabolic coupling, and macromolecular-condensate biology.

## 3 Effects of the ageing immune microenvironment on macrophage states

### 3.1 Features of the ageing-associated immune microenvironment

Ageing remodels the joint immune microenvironment through the accumulation of senescent cells and their context-dependent SASP (46). Senescent joint-resident cells and immune cells interact bidirectionally, altering both inflammatory tone and tissue repair (47). OA samples show increased infiltration of macrophages with M1-like features and higher CD86 expression in synovium (48, 49). Omics-based analyses also link OA-associated biomarkers to CD8+ T cells, memory B cells, and other immune populations, indicating that macrophage dysfunction develops within a broader, multicellular immune network (36).

Inflammageing provides a chronic low-grade inflammatory background in which innate immune pathways remain persistently engaged (48). In OA, NF-κB, MAPK, and NLRP3 signalling can converge on macrophage state. Oleamide, for example, activates the NLRP3 inflammasome and increases IL-1β production, whereas excessive NLRP3 activation in synovial macrophages amplifies local cytokine release (50, 51). Sustained activation damages cartilage and bone directly while also weakening the resolution and repair processes required to restore joint homeostasis.

### 3.2 Phenotypic and functional changes in senescent macrophages

In OA, macrophages can acquire senescence-like states characterised by phenotypic heterogeneity and functional decline that are not captured by the M1/M2 framework (52). A prominent defect is reduced phagocytic competence, particularly impaired efferocytosis (53). Persistent stress responses and SASP-like mediator production may then sustain inflammation and tissue degeneration (54). IL-1β from synovial cells can alter chondrocyte phenotype, and abnormal communication between senescent macrophages and neighbouring cells further reinforces a pro-degenerative microenvironment (55–57). Unlike M1/M2 polarization, which describes a functional bias, macrophage senescence emphasises persistent stress, SASP, mitochondrial dysfunction, and loss of homeostatic capacity (39, 58). In this context, functional criteria may be more informative than cell-cycle markers alone.

### 3.3 Mechanisms of macrophage reprogramming in the ageing immune microenvironment

Macrophage reprogramming in the ageing joint is multilayered, but immunometabolism is a central component. Disturbed lipid handling, mitochondrial dysfunction, and excess ROS provide a metabolic basis for altered macrophage function (59). Low-dose radiotherapy has been reported to shift synovial macrophage readouts towards an M2-like profile and improve pain and cartilage injury in OA rats (60). Whether this effect reflects stable reprogramming, transient cytokine suppression, or changes in macrophage recruitment remains to be established.

Transcriptional control and intercellular communication also shape macrophage state. Exosomes from hypoxia-preconditioned cartilage progenitor cells suppress NF-κB activity and promote anti-inflammatory macrophage responses (61). Substrate stiffness modifies chondrocyte–macrophage communication through exosomes, illustrating how mechanical and vesicular signals intersect (62). Nanomedicines carrying kartogenin and CA9 siRNA can reduce CA9 expression while promoting M2-like macrophage features (63). Myeloid-derived suppressor cells add further complexity because they can contribute to inflammation yet also promote Arg-1-associated M2-like responses (64). These examples support a network model in which transcriptional, metabolic, mechanical, and cell–cell signals jointly determine macrophage function.

### 3.4 Contribution of the ageing immune microenvironment to OA pathology

**Figure 3:**
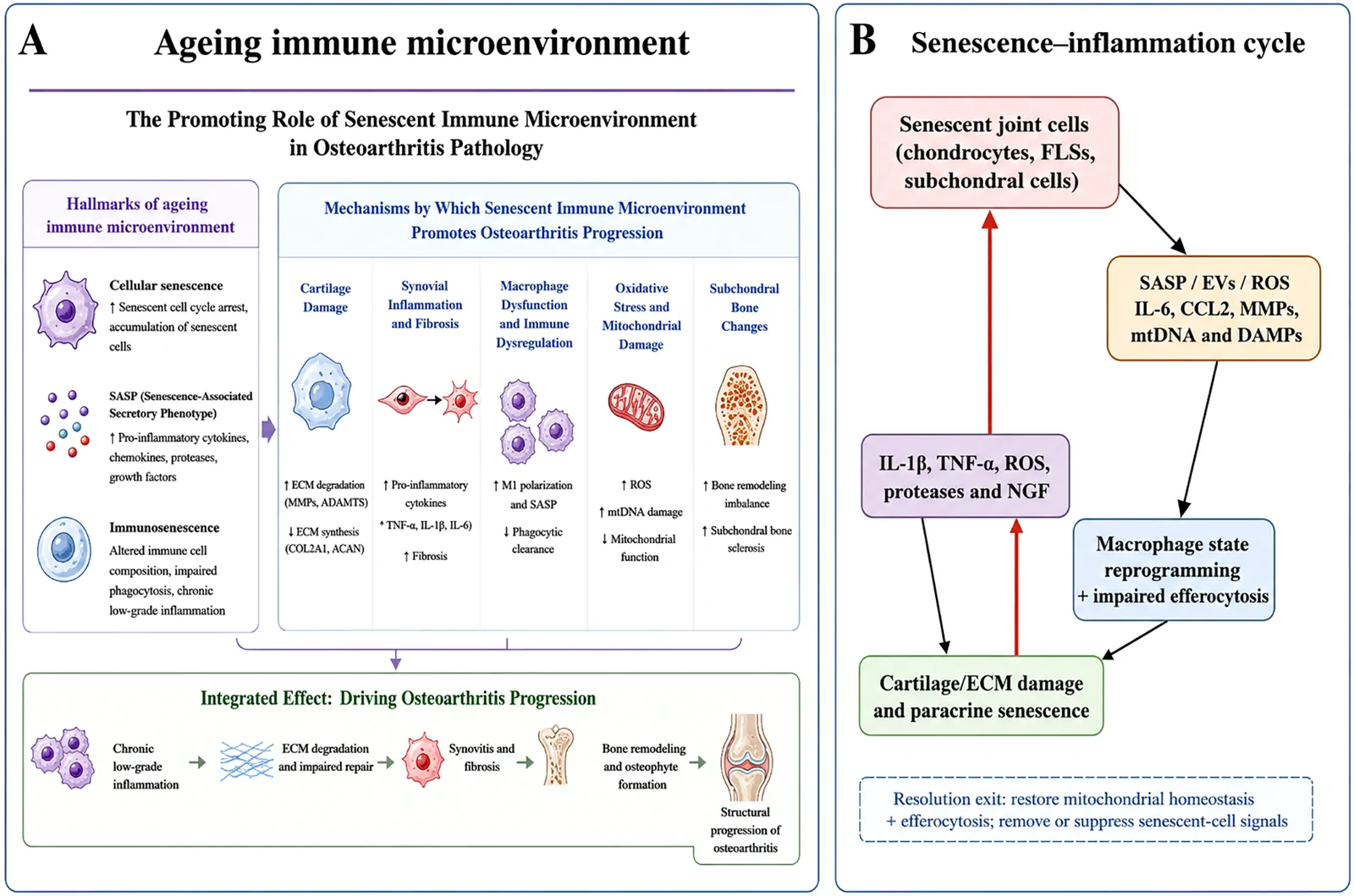
Ageing immune microenvironment and the senescence–inflammation cycle in OA. **(A)** Senescent cells, SASP, immunosenescence, oxidative and metabolic stress, and intercellular interactions drive pathological changes in cartilage, synovium, and subchondral bone. **(B)** Senescent joint-resident cells release SASP factors, EVs, ROS, mitochondrial DNA, and damage-associated molecular patterns that recruit and reprogramme macrophages. Reprogrammed or senescent-like macrophages then release cytokines, ROS, proteases, and neurotrophic factors, thereby amplifying tissue injury and paracrine senescence; impaired phagocytosis and efferocytosis further limit repair. This schematic diagram was drawn by the author using BioRender (https://app.biorender.com) and polished by artificial intelligence.

Age-related cellular senescence, SASP, and immunosenescence converge to promote joint degeneration (**Figure 3**) (65). IL-1β-treated chondrocytes express SASP factors, activate NF-κB, and increase ECM degradation, whereas replicatively senescent OA chondrocytes upregulate IL-1α, IL-1β, IL-6, and CCL2 (66, 67). The consequences of immune modulation are not uniformly beneficial: early intra-articular triamcinolone can produce short-term anti-inflammatory effects yet sustain synovial macrophage numbers and aggravate osteophyte formation in mice (68). This finding cautions against equating transient cytokine suppression with long-term structural benefit.

The ageing immune microenvironment affects the entire joint rather than cartilage alone. Synovitis and structural bone changes can accelerate disease progression, and senescence markers such as ganglioside GD3 and ST8SIA1 are increased in cartilage, synovium, and subchondral bone (69, 70). In chondrocytes, inflammatory stress preferentially induces p16 and SASP expression, whereas oxidative stress increases ROS and p21 while suppressing proliferation and glycosaminoglycan synthesis (71). Mitochondrial dysfunction is another shared feature; mitophagy limits damage by removing dysfunctional mitochondria (72). Ageing synovial fibroblasts may also acquire pro-inflammatory and pro-fibrotic phenotypes (73). These changes interact across tissues, producing a joint-wide environment that sustains inflammation and impairs repair.

## 4 Molecular mechanisms of macrophage reprogramming in osteoarthritis

### 4.1 SASP-driven transcriptional and epigenetic remodelling

The SASP is not a uniform inflammatory mixture but a context-dependent set of signals that can alter macrophage recruitment, transcription-factor activity, enhancer accessibility, and metabolic demand. Evidence from OA includes fibroblast-like synoviocyte-derived exosomal control of macrophage glycolysis, S100A12-driven inflammatory activation and inflammation–senescence crosstalk, and chondrocyte–macrophage feedback involving lysyl oxidase-like 1 and SASP-related signals (25, 27, 57, 73, 74). Repeated exposure could therefore shift macrophages from an acute, reversible response towards a state in which inflammatory programmes remain permissive after the initiating signal declines. However, direct maps linking defined SASP components to enhancer remodelling in macrophages from aged human OA tissue are still lacking. This distinction separates a plausible mechanism from one already demonstrated in OA.

Evidence from trained innate immunity provides a mechanistic framework for such persistence. Monocyte-to-macrophage differentiation and repeated innate stimulation can install H3K4me3- and H3K27ac-associated chromatin changes at inflammatory and metabolic loci (75). In OA, this framework predicts that recurrent SASP exposure could prime exaggerated responses to subsequent mechanical injury or matrix fragments. Testing the prediction will require paired single-cell transcriptomic and chromatin-accessibility data, defined exposure histories, and functional restimulation assays rather than marker co-expression alone.

### 4.2 Mitochondrial dysfunction, immunometabolism, and trained innate memory

Mitochondria integrate redox stress, metabolite availability, inflammasome activation, and phagocytic competence. Damaged mitochondria generate ROS and release mitochondrial DNA, activating NLRP3 and cGAS–STING signalling. Succinate, itaconate, and altered NADH oxidation act as immunometabolic signals, while HIF-1α/mTOR-dependent glycolysis can support trained inflammatory responses (75–77). Itaconate can also suppress STING/NF-κB signalling and promote M2-like macrophage responses in experimental OA, illustrating that individual metabolites can redirect this network (76). Exosomes derived from inflammatory fibroblast-like synovial cells promote macrophage glycolysis through HIF-1α-related mechanisms (73). While OA immune memory mesenchymal stromal cell exosomes can restore the mitochondrial ND3/NADH-CoQ axis, reduce ROS, and improve oxidative phosphorylation (78). Senescent synovial macrophages show mitochondrial injury and defective efferocytosis, directly linking organelle dysfunction to failed resolution (79).

These observations support—but do not yet prove—a trained-immunity model for aged OA. Under this model, repeated SASP and metabolic stress induce durable chromatin-metabolic coupling, so that a later injury elicits disproportionate inflammatory output. The model should be tested by washout/restimulation experiments and by determining whether interventions that normalise mitochondrial quality also erase persistent enhancer accessibility. The ROS produced by stressed chondrocytes can reasonably reprogram adjacent macrophages through oxidized mitochondrial DNA, metabolites, or exosomal vesicle (EV) cargoes. However, the direct causal evidence for this specific chondrocyte-to-macrophage pathway in osteoarthritis of elderly patients is still incomplete (80).

### 4.3 Molecular cross-talk between senescent joint-resident cells and macrophages

Senescent chondrocytes, synovial fibroblasts, subchondral-bone cells, and macrophages form a reciprocal communication network. Chondrocyte-derived IL-6 and CCL2 activate or recruit macrophages, while MMPs and aggrecanases expose ECM fragments that reinforce pattern-recognition signalling. Inflammatory fibroblast-like synoviocyte-derived exosomes increase macrophage HIF-1α-dependent glycolysis, and senescent synovial intimal fibroblasts promote inflammatory polarization through ANGPTL4–α5β1 signalling (72, 73). Conversely, macrophage-derived IL-1β, TNF-α, ROS, and EV-associated miR-155-5p induce chondrocyte catabolism, suppress autophagy, and promote senescence (18, 19, 81). Single-cell analysis of bone-marrow lesions further suggests communication between senescence-enriched monocytes and damaged cartilage across the osteochondral unit (82). Senescence, metabolic disturbance, and inflammation are therefore coupled processes that can initiate and amplify one another through ligand–receptor, vesicular, redox, and matrix-mediated routes.

### 4.4 Macromolecular condensates and gene-regulatory memory

Phase separation is a plausible biophysical mechanism through which transient signals could be concentrated into persistent gene-regulatory programmes. BRD4/MED1-rich condensates assemble at super-enhancers, and DNA-induced cGAS condensation facilitates innate immune signalling (83, 84). No direct evidence yet demonstrates pathogenic condensate dynamics in OA macrophages, however. This concept should therefore be treated as a testable hypothesis, to be examined by live-cell imaging, perturbation of condensate-forming domains, and spatial proteomics.

### 4.5 The senescence-inflammation cycle and failure of resolution

The senescence–inflammation cycle is a feedback system rather than a linear sequence (**Figure 3B**). Senescent joint-resident cells release SASP cytokines, CCL2, proteases, EVs, and redox signals that recruit and reprogramme macrophages. In turn, macrophages produce IL-1β, TNF-α, ROS, proteases, and nerve growth factor, promoting ECM damage, nociceptive sensitisation, and further cellular senescence. ECM fragments and mitochondrial danger signals then reactivate macrophage pattern-recognition pathways. Failed efferocytosis removes a key route out of this loop, allowing apoptotic and senescent-cell material to accumulate (57, 72, 74, 79, 85). Conversely, type 2 cytokine signalling and resolvin D1 can reduce senescence-associated macrophage features. These studies suggest that restoring resolution and mitochondrial competence may yield more durable benefit than non-specific inflammatory suppression.

## 5 Therapeutic strategies for osteoarthritis based on macrophage state reprogramming

### 5.1 Conventional approaches targeting M1/M2 polarization

**Figure 4:**
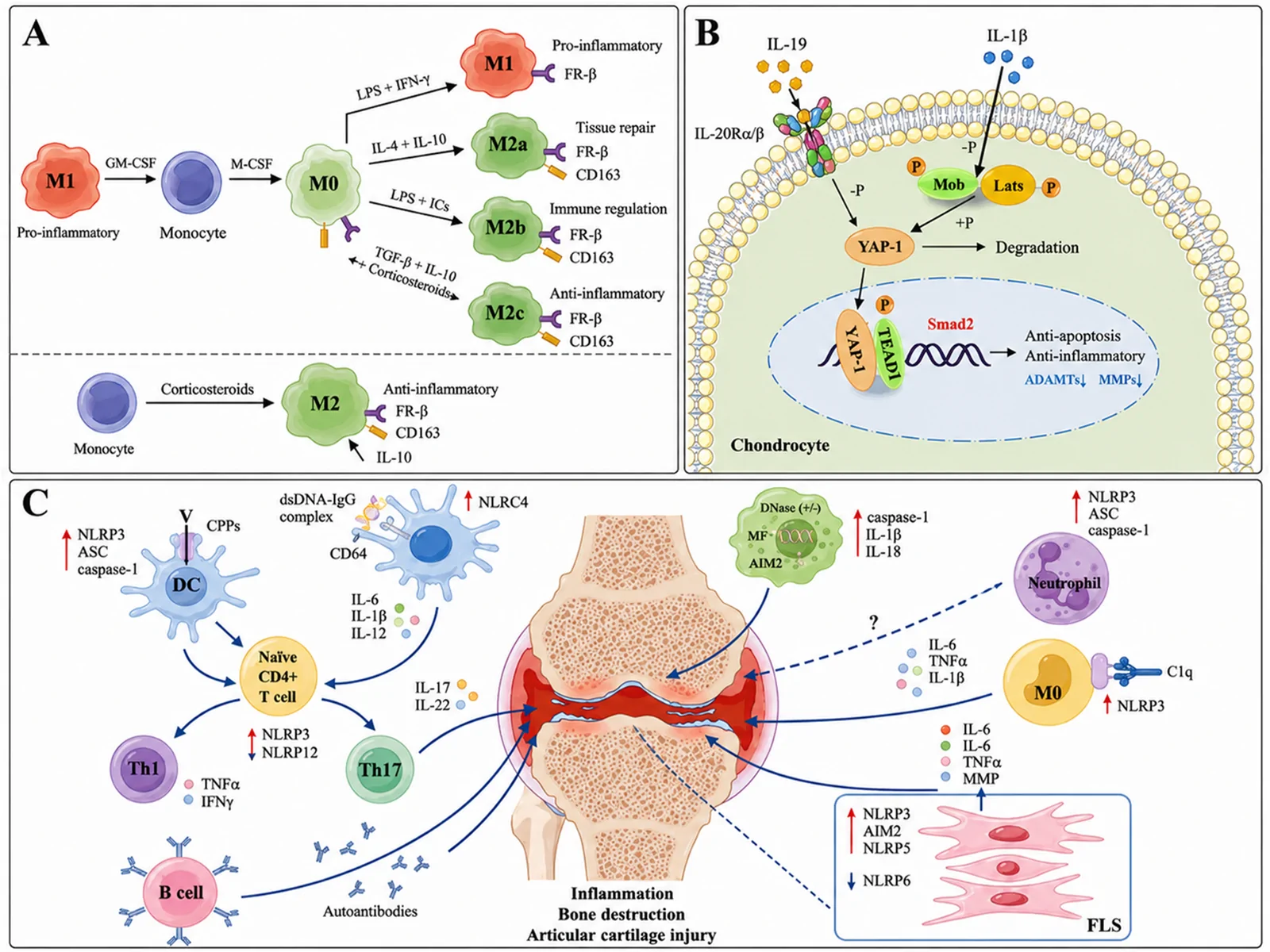
Macrophage signalling modules in OA. **(A)** Monocyte differentiation under GM-CSF, M-CSF, type 2 cytokines, and glucocorticoids, including folate receptor-β-positive anti-inflammatory macrophages (Reproduced with permission from (11). Copyright 2022, SAGE Publications Inc.). **(B)** M2 macrophage-derived IL-19 modulates the MOB1/LATS–YAP1 axis in chondrocytes, thereby limiting inflammation, apoptosis, and matrix degradation (88). **(C)** Injury- and mitochondria-derived signals activate an inflammasome-centred network that links macrophages and other immune cells to synovitis, cartilage catabolism, and subchondral remodelling (Reproduced with permission from (96). Copyright 2022, Dove Medical Press Ltd.).

Conventional approaches include non-steroidal anti-inflammatory drugs (NSAIDs), glucocorticoids, cytokine modulation, and agents that alter selected M1/M2 readouts. NSAIDs reduce macrophage-associated inflammatory activity but do not specifically reset macrophage state (86). Glucocorticoids can favour folate receptor-β-positive anti-inflammatory differentiation *in vitro* (**Figure 4A**), yet end-stage OA synovial macrophages may occupy a tolerised transcriptional state with weak corticosteroid responsiveness (11, 87). Moreover, suppressing one cytokine may leave upstream damage sensing, metabolic dysfunction, and multicellular crosstalk intact (**Figure 4C**). These limitations help explain why symptomatic anti-inflammatory effects do not necessarily translate into durable structural modification.

More selective molecular interventions identify potentially tractable nodes. M2 macrophage-derived IL-19 reduces IL-1β-induced chondrocyte inflammation and apoptosis by modulating Hippo/YAP1 signalling (**Figure 4B**) (88). α-Defensin-1 promotes repair-associated macrophage responses (89), Annexin A5 enhances lysosomal degradation and suppresses inflammatory polarization (90), and betulinic or carnosic acid modulates the GSK3β/NF-κB/CCL20 or Nrf2/NF-κB axis (43, 59). These studies establish biological plausibility, but efficacy should be judged by restored efferocytosis, stable state change, pain relief, and structural preservation.

### 5.2 Emerging macrophage reprogramming technologies

**Figure 5:**
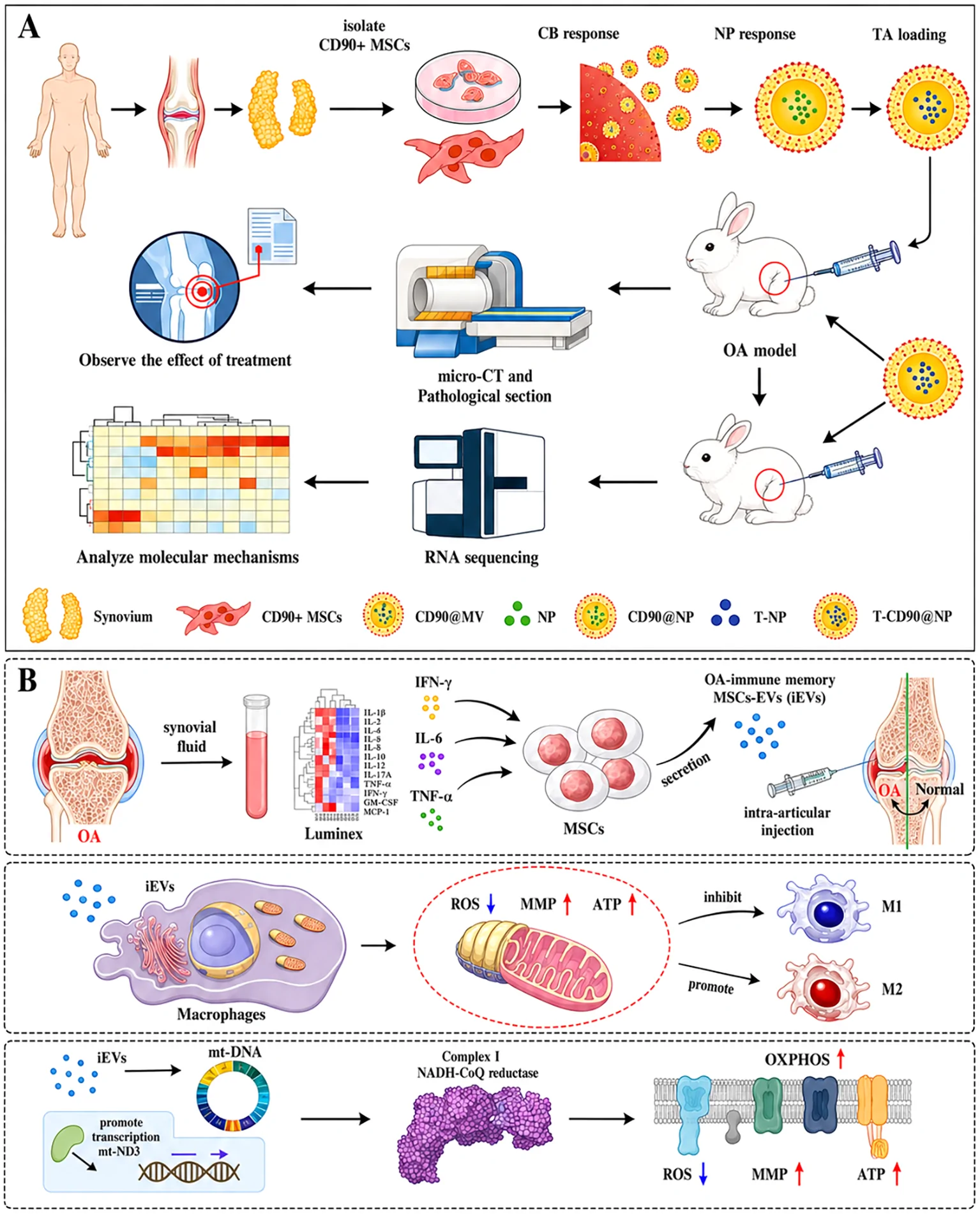
MSC-based delivery systems for OA. **(A)** CD90+ synovial mesenchymal stromal-cell-derived microvesicles coat triamcinolone-loaded nanoparticles, improving local delivery and promoting repair-associated macrophage responses (Reproduced with permission from (92). Copyright 2022, BioMed Central.). **(B)** OA immune-memory MSC-derived EVs reprogramme the macrophage mt-ND3/NADH-CoQ axis, reduce ROS, support oxidative phosphorylation, and suppress inflammatory output (Reproduced with permission from (78). Copyright 2025, BioMed Central.).

Cell, gene, and EV-based approaches can deliver multiple regulatory signals simultaneously. TissueGene-C generates an M2-like-dominant immune environment in a rat monoiodoacetate model (91). Engineered or conditionally primed mesenchymal stromal-cell EVs can transfer microRNAs, proteins, and mitochondrial-axis-associated cargo; OA immune-memory MSC-EVs, for example, reprogramme the macrophage mt-ND3/NADH-CoQ axis (**Figure 5B**) (78). Their principal advantage is the potential to coordinate metabolism, inflammation, and intercellular communication. Their main liabilities are cargo heterogeneity, batch-to-batch variability, incomplete definition of mechanism of action, and manufacturing reproducibility.

**Figure 6:**
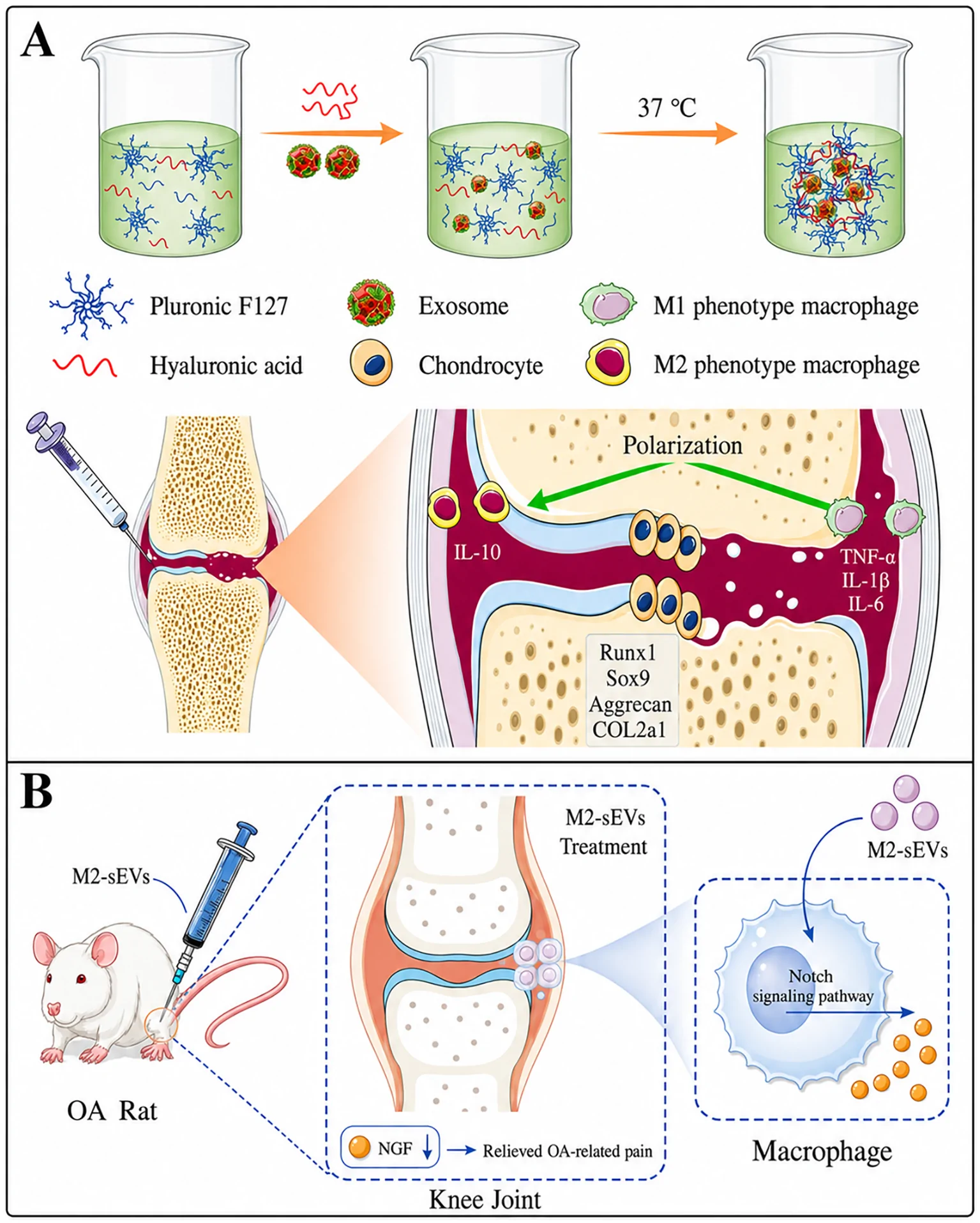
Exosome-based local therapy in OA. **(A)** Primary chondrocyte-derived exosomes achieve sustained intra-articular delivery in an F127–hyaluronic acid thermosensitive hydrogel and promote cartilage-protective macrophage responses (Reproduced with permission from (93). Copyright 2022, Springer Singapore.). **(B)** M2 macrophage-derived small EVs modulate Notch signalling in synovial macrophages, suppress macrophage-derived nerve growth factor, reduce macrophage–neural inflammatory crosstalk, and alleviate OA-related pain (Reproduced with permission from (100). Copyright 2026, Taylor & Francis Group.).

Nanomedicines and biomaterials can increase local exposure and couple release to pathological cues. CD90+ synovial mesenchymal stromal-cell-derived microvesicle-coated nanoparticles deliver triamcinolone and promote repair-associated macrophage responses in preclinical OA (**Figure 5A**) (92). Primary chondrocyte-derived exosomes embedded in a thermosensitive F127–hyaluronic acid hydrogel improve local retention and promote cartilage-protective macrophage responses (**Figure 6A**) (93). Curcumin-loaded thermosensitive hydrogels and ROS-, cytokine-, or matrix-degradation-responsive systems extend this principle (94, 95). Nevertheless, claims of targeting require quantitative biodistribution and cell-specific uptake data, particularly in aged joints where synovial permeability, lymphatic clearance, fibrosis, and phagocyte composition differ from those in young experimental models.

### 5.3 Senescence-targeted therapy and immune microenvironment modulation

Senolytics eliminate selected senescent cells, whereas senomorphics attenuate harmful SASP without necessarily killing the cell. Their rationale differs from direct M1-to-M2 repolarisation because they target an upstream source of repeated macrophage stimulation rather than imposing a downstream marker profile (80, 85, 97, 98). This distinction may be especially relevant in aged joints, where persistent SASP, matrix damage, and mitochondrial danger signals can rapidly reverse a transient M2-like shift. The advantage is therefore potentially greater durability, not universal superiority. Senolytics may remove repair-competent or beneficially senescent cells, while senomorphics may require sustained exposure and can suppress protective secretory functions. Both approaches must account for heterogeneous senescent-cell survival pathways, tissue compartment, and disease stage.

### 5.4 Comparative assessment and combination strategies

No single intervention is likely to correct the full ageing-associated network. Direct repolarisation can act rapidly, but the effect may be unstable if SASP and metabolic stress persist. Senolytics reduce upstream cell burden but may miss non-senescent inflammatory sources and introduce tissue-specific toxicity. Senomorphics are reversible and may preserve cell viability, yet they often require continued exposure. Metabolic reprogramming can restore mitochondrial fitness, efferocytosis, and state plasticity, whereas broad inhibition of glycolysis or glutamine use may impair host defence. Therapeutic choice should therefore be based on the dominant pathological state, anatomical compartment, and disease stage rather than on a universal M1/M2 ratio.

Combination regimens should be mechanism-based and tested with explicit temporal logic. One hypothesis is to reduce sustained SASP with a senolytic or senomorphic before restoring mitochondrial function or efferocytosis, thereby improving the capacity of macrophages to exit the inflammatory loop. For example, in preclinical osteoarthritis models, coated berberine/V-9302 nanoparticles can simultaneously inhibit HIF-1α/GLUT1-related glycolysis and glutamine uptake (99). A polymeric pCQ/SOD platform restores mitochondrial homeostasis and efferocytosis in senescent synovial macrophages, while dual senolytic–senomorphic biomimetic nanovesicles combine two upstream interventions (79, 85).

### 5.5 Translational bottlenecks and clinical development

Therapeutic selection in ageing OA should be guided by the dominant upstream disturbance rather than by the M1/M2 ratio alone. Direct repolarisation may quickly reduce inflammatory output, but persistent SASP, metabolic stress, and mitochondrial damage can drive relapse. Senolytics may remove chronic sources of stimulation, whereas senomorphics may preserve cell viability while reducing harmful secretion. These benefits must be balanced against senescent-cell heterogeneity, depletion of repair-competent cells, limited intra-articular retention, off-target immune modulation, and uncertain long-term safety. A combination of senescence-targeted treatment with metabolic reprogramming, efferocytosis restoration, EV delivery, or responsive biomaterials may therefore be more durable than a single-node intervention, but this premise requires direct head-to-head and sequencing studies (80, 85, 97–99) (**Table 1**).

**Table 1:** Therapeutic strategies targeting macrophage state reprogramming in osteoarthritis.

Therapeutic category	Agent/platform	Research level and models	Mechanism	Effects	Ref.
Conventional anti-inflammatory therapy	Triamcinolone	Preclinical; human monocyte-derived macrophages and OA animal models.	Modulates macrophage polarization; end-stage OA synovial macrophages may exhibit a tolerised state with limited corticosteroid responsiveness.	Provides short-term anti-inflammatory activity; limited long-term structural benefit may reflect reduced transcriptional responsiveness.	(11, 87)
Natural compounds targeting inflammatory signalling	Betulinic acid	Preclinical; macrophage/chondrocyte models and OA animal models.	Suppresses M1-like features and promotes an M2-like phenotype, partly through inhibition of NF-κB signalling.	Reduces inflammatory macrophage activation and may attenuate OA pathology.	(43)
Natural compounds targeting redox and inflammatory pathways	Carnosic acid	Preclinical; macrophage polarization models and OA animal models.	Enhances Nrf2 signalling and inhibits NF-κB; suppresses LPS-induced M1-like polarization and enhances IL-4-induced M2-like polarization.	Promotes anti-inflammatory macrophage skewing and may protect joint tissues.	(59)
Engineered extracellular vesicles	Conditioned OA immune-memory mesenchymal stromal cell-derived extracellular vesicles	Preclinical; OA immune-memory MSC-derived EV platform tested in cellular and animal OA models.	Deliver multi-component cargo that restores the macrophage mt-ND3/NADH-CoQ axis and coordinates metabolic and inflammatory reprogramming.	Reduce ROS, support oxidative phosphorylation, and promote repair-associated macrophage function; cargo and manufacturing heterogeneity remain concerns.	(78)
Conventional anti-inflammatory therapy	Non-steroidal anti-inflammatory drugs (NSAIDs)	Preclinical; human OA synovial fluid immune cells/macrophage studies and inflammatory cell models.	Suppress macrophage-associated inflammatory activity without directly inducing a defined macrophage-state transition.	Provide symptomatic anti-inflammatory effects, with potential indirect modulation of the joint immune microenvironment.	(86)
Cytokine-based modulation	M2 macrophage-derived IL-19	Preclinical; macrophage/chondrocyte inflammatory models and OA animal models.	Suppresses Hippo signalling and YAP1 phosphorylation, reducing IL-1β-induced chondrocyte inflammation and apoptosis.	Attenuates inflammatory and apoptotic responses in chondrocytes.	(88)
Bioactive peptides	α-Defensin-1	Preclinical; macrophage polarization models and OA animal models.	Promotes an M1-to-M2-like macrophage shift and indirectly supports chondrocyte protection.	Enhances anti-inflammatory macrophage responses and may reduce cartilage injury.	(89)
Metabolic and molecular regulators	Annexin A5	Preclinical; macrophage/chondrocyte inflammatory models and OA animal models.	Inhibits M1-like polarization by promoting lysosomal degradation.	Protects chondrocytes from necrosis and apoptosis.	(90)
Cell and gene therapy	TissueGene-C	Preclinical; OA-related macrophage and chondrocyte models.	Induces an M2-like macrophage-dominant immune microenvironment in experimental OA.	Reduces OA pathology in preclinical models.	(91)
Biomimetic nanoparticles	CD90+ synovial mesenchymal stromal cell-derived microvesicle-coated triamcinolone nanoparticles	Preclinical; rat monoiodoacetate-induced OA model.	Enhance local delivery and injured-cartilage uptake while promoting IL-10-associated repair responses.	Provide anti-inflammatory and cartilage-protective effects in preclinical OA models; quantitative targeting in aged joints remains to be established.	(92)
Exosome-based therapy	Primary chondrocyte-derived exosomes	Preclinical; synovial MSC-derived microvesicle delivery system evaluated in OA models.	Promote a transition from inflammatory to repair-associated macrophage states within a sustained-release hydrogel.	Reduce inflammatory responses and support cartilage-protective macrophage functions.	(93)
Nanomedicine-based macrophage modulation	Nanodrug carriers	Preclinical; primary chondrocyte-derived exosomes, macrophage models, and OA animal models.	Enable targeted regulation of macrophage metabolism and polarization.	Provide controlled or microenvironment-responsive immunomodulation.	(94)
Biomaterial-assisted delivery	Curcumin-loaded thermosensitive hydroxybutyl chitosan hydrogel	Preclinical; nanoparticle-based macrophage modulation studies in OA models.	Promotes M2-like macrophage polarization within the joint microenvironment.	Attenuates cartilage degeneration and inflammation in OA models.	(95)
Senescence-targeted therapy	Senolytics and senomorphics	Preclinical; OA cellular and animal models.	Eliminate selected senescent cells or suppress harmful SASP, thereby reducing persistent upstream stimulation of macrophages.	May provide more durable effects than phenotype-only repolarization, but efficacy is limited by senescent-cell heterogeneity and potential loss of repair-competent cells.	(97, 98)
Combination strategies	Senescence-targeted agents combined with metabolic- or efferocytosis-restoring platforms	Preclinical; macrophage inflammatory and senescence-related models.	Reduce sustained SASP-driven stimulation while restoring mitochondrial fitness, efferocytosis, and macrophage-state plasticity.	May provide more durable effects than single-node repolarization; optimization of sequencing, local retention, cell-state specificity, and safety is required.	(99)
Engineered small extracellular vesicles	M2-derived small extracellular vesicles	Preclinical; macrophage/chondrocyte models and OA animal models.	Modulate Notch signalling and suppress synovial macrophage-derived nerve growth factor (NGF).	May alleviate OA pain by regulating macrophage–neural inflammatory crosstalk.	(100)
Injectable immunomodulatory biomaterials	Chondroitin sulfate methacrylate-based injectable hydrogel microspheres	Preclinical; artificial M2 macrophage-like platform evaluated in OA models.	Induce macrophage polarization toward an anti-inflammatory M2 phenotype.	Provide local modulation of the OA immune microenvironment.	(101)
Artificial macrophage-like systems	Artificial M2 macrophage-like platform composed of macrophage membrane and inflammation-responsive nanogel	Preclinical; synovial inflammation and macrophage experimental models.	Target inflamed regions, prolong local retention, and release payloads in response to inflammation.	Suppress acute inflammation and promote cartilage repair in preclinical OA models.	(102)
Repurposed or clinically used agents with macrophage effects	High-molecular-weight hyaluronic acid	Preclinical; macrophage inflammatory models.	Suppresses IL-1β-induced synovial inflammation and M1-like polarization through the GRP78–NF-κB pathway.	Reduces synovial inflammation and may contribute to macrophage-mediated immunomodulation.	(103)
Natural small molecules with defined macrophage targets	Ellagic acid	Preclinical; macrophage polarization and OA models.	Targets PTGS2/COX-2 in M1 macrophages and inhibits prostaglandin E2 production.	Exerts anti-inflammatory and chondroprotective effects.	(104)

Arg-1, arginase-1; COX-2, cyclooxygenase-2; GRP78, glucose-regulated protein 78; IL, interleukin; LPS, lipopolysaccharide; MMPs, matrix metalloproteinases; NF-κB, nuclear factor-κB; NGF, nerve growth factor; NSAIDs, non-steroidal anti-inflammatory drugs; OA, osteoarthritis; PTGS2, prostaglandin-endoperoxide synthase 2; ROS, reactive oxygen species; SASP, senescence-associated secretory phenotype; YAP1, Yes-associated protein 1; OA, Osteoarthritis.

Despite extensive preclinical evidence, macrophage reprogramming has not been validated as a disease-modifying OA therapy. Major barriers include rapid joint clearance, heterogeneous uptake by resident and infiltrating phagocytes, incomplete state specificity, systemic immunosuppression, and uncertain long-term effects on resident macrophages. Senescent-cell heterogeneity adds another layer: p16-, p21-, DNA-damage-, mitochondrial-, and SASP-dominant populations may not respond to the same agent. Most young, surgically induced rodent models also underrepresent immunosenescence, multimorbidity, sex differences, obesity, and the chronicity of human OA. Translation will require aged and metabolically relevant models, human explant validation, quantitative biodistribution, scalable manufacturing, and predefined safety criteria for infection and impaired repair.

Patient stratification will be essential. Composite biomarkers should integrate tissue or synovial-fluid senescence modules, macrophage-state signatures, metabolic markers, and imaging-defined lesion location rather than rely on a single cytokine or surface marker. Early trials should pair pain and structural outcomes with pharmacodynamic evidence of target engagement, such as reduction of a SASP module, restoration of efferocytosis, or alteration of a spatially defined macrophage state. State-specific delivery—for example, folate-receptor-targeted nanoparticles or engineered small EVs that suppress macrophage-derived nerve growth factor through Notch modulation (**Figure 6B**)—may reduce systemic exposure (97, 100).

## 6 Conclusions

Macrophage research in OA is moving from a binary polarization narrative towards a state-reprogramming framework. The M1/M2 axis remains a useful descriptive dimension, but it cannot capture cellular origin, exposure history, senescence, metabolic fitness, enhancer memory, efferocytosis, or spatial context. In aged joints, senescent-cell SASP, mitochondrial danger signals, ECM fragments, and multicellular communication create a self-reinforcing senescence-inflammation cycle. Macrophages are both recipients and amplifiers of this network, and their pathological activity may persist even when conventional M1 markers decline.

Therapeutic goals should therefore move beyond simply increasing M2-labelled cells. Removing or suppressing upstream senescent-cell signals, restoring mitochondrial quality and efferocytosis, and delivering state-specific regulators locally may provide more durable control. Senolytics, senomorphics, metabolic agents, extracellular vesicles, and responsive biomaterials each address different nodes and have distinct risks. Rational combinations should be selected according to disease stage and dominant state, tested in aged and metabolically relevant models, and evaluated for infection susceptibility, wound repair, pain, structural outcomes, and durability after treatment withdrawal.

Priority areas are: (i) spatial transcriptomic and multiplex-imaging maps of macrophage trajectories across synovium, cartilage, infrapatellar fat pad, bone-marrow lesions, and subchondral bone in aged human OA; (ii) paired single-cell transcriptomic, chromatin-accessibility, proteomic, and metabolic measurements that distinguish transient activation from trained or senescent states; (iii) lineage-aware human explant and organoid models that preserve mechanical loading and multicellular communication; (iv) composite senescence–macrophage biomarkers for patient stratification; and (v) biomarker-enriched early-phase trials with pharmacodynamic evidence of target engagement. Together, these steps could turn macrophage state reprogramming from a descriptive concept into a testable basis for precision, disease-modifying OA therapy.
